# Effect of Ambient Temperature on Daily Nebulized Asthma Hospital Visits in a Tropical City of Dhaka, Bangladesh

**DOI:** 10.3390/ijerph18030890

**Published:** 2021-01-20

**Authors:** Ayesha Ferdosi Kabir, Chris Fook Sheng Ng, Shinya Yasumoto, Taiichi Hayashi, Chiho Watanabe

**Affiliations:** 1 Department of Human Ecology, School of International Health, Graduate School of Medicine, The University of Tokyo, Tokyo 113-0033, Japan; ayesha.ferdosi@gmail.com (A.F.K.); yasumoto@isc.chubu.ac.jp (S.Y.); chiho.watanabe@nies.go.jp (C.W.); 2 School of Tropical Medicine and Global Health, Nagasaki University, Nagasaki 852-8523, Japan; 3 Center for Southeast Asian Studies, Kyoto University, Kyoto 606-8501, Japan; hayashi@cseas.kyoto-u.ac.jp; 4 National Institute for Environmental Studies, Tsukuba, Ibaraki 305-0053, Japan

**Keywords:** outdoor temperature, asthma, short-term exposure, risk assessment, delayed effect, tropical climate

## Abstract

The acute effect of temperature on asthma morbidity in Bangladesh is not well understood. As climate varies extensively in different parts of the world, the relation between temperature and asthma might also differ. We investigated the association between temperature and asthma-related hospital visits in the tropical city of Dhaka. We analyzed information from a total of 5989 asthma patients who received ambulatory care in the form of nebulized medication at the National Asthma Center in Mohakhali, Dhaka from February to November 2013. A time-stratified case-crossover study was conducted to estimate the effect of daily temperature, with consideration of delayed effects and possible confounders such as relative humidity and political strikes. An inverse association was observed between temperature and the number of hospital visits. The effect was delayed for approximately a week. A degree centigrade decrease in mean temperature (averaged across lags 0-6) was associated with an increase of approximately 4.5% (95% CI 1.5, 7.5) in all asthma visits. The association was evident in adult males but marginal in elderly males. A positive association (lag 0) was observed among adult females, whereas no association was observed among children. Strikes significantly modified the effect among the elderly. Findings suggest temperature declines affect asthma outcomes in a warm climate, and this effect can be delayed and vary by sex and age group.

## 1. Introduction

Asthma is a chronic respiratory disease affecting an estimated 300 million people worldwide, with approximately 250,000 annual deaths reported [1,2,3]. The prevalence of the disease has been rising around the world [4] and this increasing trend is particularly discernible in developing countries undergoing urbanization [5]. In Bangladesh, a national study conducted in 1999 reported a prevalence of 6.9% based on self-reported data; the study estimated that 7 million people, 4 million of whom were children, suffered from asthma-related symptoms [6]. The prevalence of clinical asthma was 3.8% in this population [1]. The disease poses as a serious public health problem in the country [7,8]. Yet, very little is known about its etiology and risk factors in Bangladesh.

Although numerous studies have examined the influence of meteorological factors, notably temperature, on the acute exacerbation of asthma, results are generally mixed. Asthma exacerbations have been associated with low temperature in London [9], Finland [10], and Shanghai [11,12]. But the association was not observed in North Carolina [13], Ottawa [14] and Delhi [15]. 

The effect of low temperature can also differ by age group. In Spain, the effect was observed in children [16], but not in adults [17]. In Tokyo, a study of ambulance-transported asthma patients reported the opposite, that asthma exacerbation was associated with low temperature in adults, but not in children [18]. A study in New York City reported a positive association between summer temperature (i.e., hot) and adult daily hospital admissions for respiratory diseases comprising mostly asthma cases [19]. A positive association was also observed among pediatric asthma patients in Detroit, Michigan [20]. In a Swedish study, different results were reported for children and adults. Low temperature was found to affect asthma in children, whereas in adults, it was high temperature [21]. Because weather and population characteristics can vary by location, heterogeneity of the findings observed thus far suggests that the relationship between temperature and asthma should be examined locally in order to identify vulnerable individuals. 

Given the lack of empirical research from Bangladesh, we conducted a study to estimate the effect of daily temperature on nebulized asthma hospital visits in the city of Dhaka. During the study period, political protests or strikes occurred frequently in the study location. These recurring strikes known as “Hartal” usually involve the restrictions of public and private transport, which can impede the mobility of a community, causing those who require medical treatment to postpone hospital visits or seek treatment elsewhere. Similar disruptions in the utilization of health care services due to political unrest have been reported in Nepal [22]. In view of this, we also examined the potential effect modification of the temperature-asthma visit association by this social phenomenon.

## 2. Materials and Methods 

### 2.1. Patients’ Data

**Figure 1:**
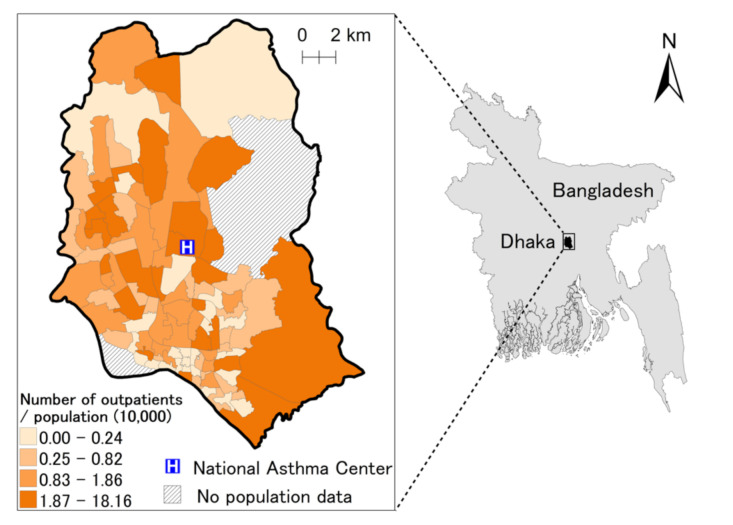
Location of the hospital and the number of outpatients (per 10,000 population), February–November 2013. Information of administrative area boundary was collected by Dhaka City Corporation.

Data on daily hospital visits were collected from the National Asthma Centre (NAC) situated in central Dhaka from February to November 2013. NAC is a specialized division of the National Institute of Disease and Chest Hospital (NIDCH) for treating respiratory diseases. NAC receives patients from different socioeconomic backgrounds from all over Dhaka and the surrounding areas. In this study, we included only patients residing within metropolitan Dhaka where meteorological data were available (Figure 1). Data were collected manually every day in the absence of an electronic data collection system. We interviewed all outpatients who visited NAC using a questionnaire after doctors completed their medical examination. We recorded the patients’ age, sex and residential address, and extracted the details of clinical diagnosis and medication from the doctors’ prescriptions. For data analysis, we selected patients who had been clinically diagnosed with asthma and who received the nebulized corticosteroid treatment (Figure S1). At NAC, nebulized corticosteroid is the rescue medication for the ambulatory care of asthma, and hence, patients receiving this treatment can be regarded as surrogates for emergency department visits for asthma attack. 

### 2.2. Ethics Approval

We obtained written informed consent from all participants, or their guardians, after explaining the study details to them before participation. The protocol for this study was approved by the National Institute of Diseases of the Chest and Hospital (NIDCH) in Dhaka, Bangladesh, and the ethics committee of the Graduate School of Medicine, The University of Tokyo, Japan.

### 2.3. Meteorological and Strike Data

We collected daily 24-h mean temperature (°C) and relative humidity (%) in accordance with previous literature [9,10,11,12,13,14,15,16,17,18,19,20,21,22]. Meteorological data were obtained from the International Centre for Diarrheal Diseases Research, Bangladesh (ICDDR, B) through an existing collaboration. The data were available at minute interval and measured by instruments installed on the rooftop of a building at ICDDR, B in Dhaka city.

To construct an indicator variable for strikes, we recorded the dates of all strike occurrences in the Dhaka area during the study period based on announcements in broadcast and print media. Strike variable was expressed as a 3-level categorical variable representing days without strikes, days with strikes, and days immediately after strikes. We distinguished the days immediately after strikes from the normal days with no strike to understand how disruptions and reopening of healthcare access could influence the association.

### 2.4. Statistical Analysis

We conducted a time-stratified case-crossover study to estimate the effect of temperature on the daily number of nebulized asthma hospital visits. The case-crossover study design is commonly used to assess the health effects of ambient exposure [13,14,23]. This method adjusts for the day-of the-week effect, seasonality, long-term trend, and time-invariant variables such as sex, occupation, education level by design. For each nebulized asthma case, we selected the same days of the week within the same month as matching control, providing three to four control days per case. Meteorological data were linked to each case and control based on dates. The distributions of mean temperature between the cases and controls were then compared [24]. Conditional logistic regression was fitted to estimate the odds ratios and 95% confidence intervals (CI) for the associations between temperature and asthma visits. We stratified our analysis by age group (children ≤18, adults 19–59 and the elderly ≥60 years old) and sex, with adjustment for relative humidity (as a linear term) and strikes. We verified the estimated effects using leave-one-out cross validation method with random omission of stratum repeated for 1000 times.

To account for the potential delayed effects of temperature, we estimated the cumulative effects by averaging the same-day temperature and its lag terms up to the previous 2 weeks (i.e., average of daily mean temperatures across lags 0 to 1, lags 0 to 2, etc.) to check the attenuation of delayed effects. To examine potential effect modification by strike events, we stratified the estimates of temperature effect based on the category of strike, that is, days unaffected by strike, strike days, or days immediately after a strike which could last for more than a day. The results are expressed as the percentage change in daily nebulized asthma visits for a degree centigrade decrease in mean temperature. All analyses were performed using R version 3.0.3 (R Foundation for Statistical Computing, Vienna, Austria).

## 3. Results

**Figure 2:**
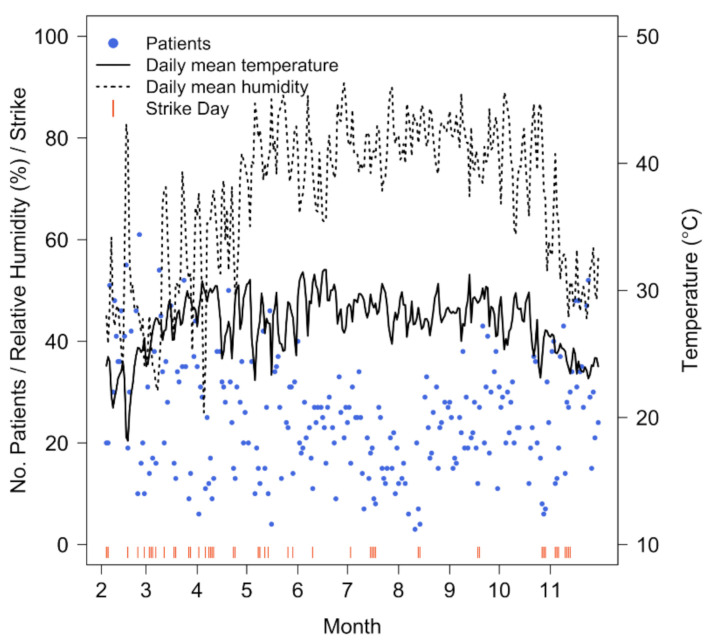
Distribution of daily nebulized asthma hospital visits (dot), mean temperature (solid line), mean relative humidity (dashed line), and strike events (short vertical bar at the bottom) near study location in Dhaka, Bangladesh, February–November 2013.

We analyzed a total of 5989 nebulized asthma patients (58% male) during the 10-month period (298 days) in 2013. Among them, 18% were children, 66% were adults and 16% were elderly. Strikes occurred for a total of 48 days and each episode lasted for approximately 1–4 days (Table 1). Excluding Fridays (Muslim holidays) and public holidays (hospital was closed), the daily number of nebulized asthma patients was lower on average when there was a strike, with a mean of 14.1 (SD: 6.3) patients versus 27.7 (SD: 10.9) patients per day when there was no strike. Figure 2 depicts the daily time series for nebulized asthma patients, mean temperature, mean relative humidity, and strikes. The daily 24-h mean temperature during the study period ranged from 18.2–31.6 °C with a median of 27.8 °C, while relative humidity ranged from 26.0–90.8% with a median of 73.4%.

**Figure 3:**
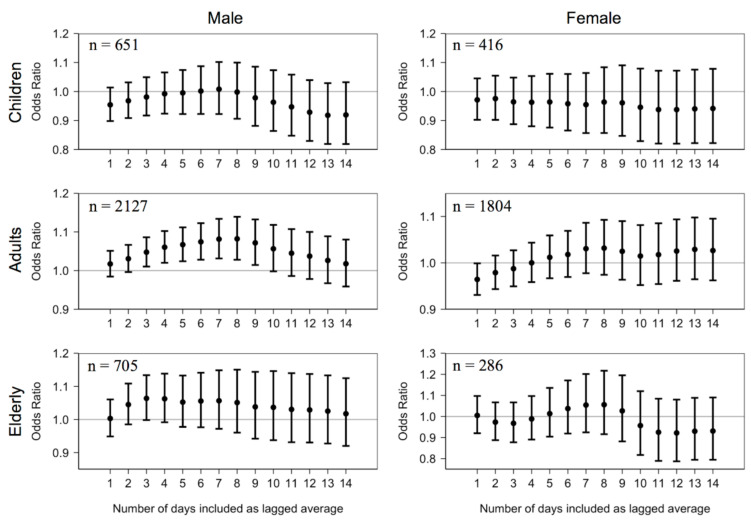
Adjusted odds ratios for the daily number of asthma patients treated with nebulized medication in relation to a 1°C decrease in daily mean temperature. Vertical lines denote the 95% confidence intervals. Temperature lags up to the previous two weeks are constrained as average.

A degree centigrade decrease in mean temperature (averaged across lags 0–6) was associated with an increase of approximately 4.47% (95% CI 1.53, 7.50) in daily nebulized asthma patients (Table 2). The estimated effect was delayed for approximately a week and observed mainly in adult males, who documented an 8.22% increase (95% CI 2.81, 13.92) in daily nebulized cases for a degree centigrade decrease in mean temperature averaged across lags 0–7 (Figure 3). Among the elderly males, we observed an increase of 6.38% (95% CI −0.19, 13.39) with a borderline statistical significance (*p* = 0.057) and a shorter delayed effect (lags 0–2). No association was observed in children or females.

When stratified by the indicator of strikes, the cumulative effect of temperature remained significant among adults at 7.21% (95% CI 3.03 to 11.56) in the absence of strikes (Table 3). This effect was observed among adult males with a lag structure lasting for about a week (Figure S2). We did not find any association in adults on days immediately after a strike. Among the elderly patients, the effect of temperature decline was significant on days immediately after a strike. A degree centigrade decrease in mean temperature (averaged lags 0–4) was significantly associated with an increase of approximately 15.65% (95% CI 1.23 to 32.12) in daily visits (Table 3). The association started from the first day (lag 0) in elderly males (Figure S2). Adult females showed an opposite association at lag 0 when there was no strike (Figure S2). We did not observe any association in children.

## 4. Discussion

This study found an inverse association between temperature and asthma exacerbations in the tropical city of Dhaka. A degree centigrade decrease in mean temperature (average of lags 0–6) was associated with an increase of approximately 4.5% in the daily number of nebulized asthma visits (Table 2). This effect was seen primarily in adult males. Similar associations have been reported in cooler climates. For example, in Finland, the daily number of emergency room visits due to asthma attacks has been negatively associated with temperature [10]. Inverse associations have also been observed among the elderly in London [9], and among children in Spain [16] and Sweden [21]. In subtropical regions, adverse effects of temperature decrease have been reported in adults and children in Shanghai [11,12]. In Tokyo, low temperature was associated with asthma exacerbations in adults, but not in children [18]. The inverse association was not observed in Delhi [15] despite having a comparable meteorological condition. A study in the subtropical Mediterranean climate of Athens [25] also did not report such an association. Nonetheless, results of the current study suggest that in the warm climate of Dhaka (relatively warmer than Shanghai and Tokyo), a drop in temperature can exacerbate asthma outcomes. This effect is delayed for about a week (Figure 3), which is shorter than the delayed cold effect observed among adult patients in Shanghai (extending from lags 14–30) [12]. The shorter and more immediate temperature effect observed in the current study is probably due to the warmer cold season in Dhaka. Inconsistencies with the findings from Delhi and Athens are possibly due to differences in methodology (i.e., the two studies analyzed monthly aggregated data and did not examine the acute effects of temperature shorter than a month). More recently, a systematic review of 26 studies reported evidence of asthma risk associated with temperature drop, particularly in children and populations in lower latitudes (warmer) [26].

The specific biological mechanisms through which low temperature induces asthma in a warm tropical climate are not known. Temperatures, both high and low, have been shown to aggravate asthma in a mouse model [27]. In temperate climates, it has been suggested that cold temperature might precipitate asthma through reductions in lung function, bronchoconstriction, inflammation, mucous hypersecretion and other responses that increase susceptibility particularly in those already affected by the condition [28,29,30,31]. Temperature declines that often precede the onset of respiratory tract infections [30] might also provide a clue to understand how cold might trigger asthma [31]. Other indoor and outdoor environmental triggers of asthma such as air pollutants, mold, pest and smoke have been identified [32], but the role of these factors in warmer climates should be elucidated further. A plausible explanation for the current findings is that populations in tropical climate might be more adapted to heat than cold as they have more protective measures against high temperatures. In Bangladesh, the use of cooling devices is more common compared to heating devices. In addition, houses are built with ventilation in mind to allow natural cooling using outdoor air, and for this reason, they are less insulated against cold, offering some explanations for the observed vulnerability to low temperature. Similar thermal adaptability to warm environments has been documented in other tropical locations. A study in Malaysia showed that despite the hot and humid local climate, certain populations might still perceive a cool outdoor temperature, indicating possible vulnerability to low temperatures relative to the local environment [33]. Adaptation to warm climates could also occur physiologically as suggested by a study in Matlab, Bangladesh that reported the evidence of excess mortality associated with cold, but not heat [34].

In the current study, the effects of low temperature on asthma exacerbations were observed predominantly in adult males aged 19–59 years old. A possible explanation for this is the variation of exposure across age and sex subgroups. The observed heterogeneous results between adults and children have also been reported elsewhere [16,17,18,21]. In Bangladesh, the male population in this age bracket makes up the main working population and generally engages in a greater amount of outdoor activity. It is possible that the personal exposure of this subgroup is more correlated with outdoor temperature compared to children, women and the elderly who spend more time indoors at home or in school. Further studies are needed to determine if variations in the temperature-asthma association observed in different subpopulations can be explained by differences in mobility patterns, occupation, or other factors. 

We observed a marginal temperature effect among the elderly males in our study. A previous study in London reported an association in the elderly population aged 65 years and above [9], where a degree centigrade decreased in temperature (below 5 °C) was associated with 12.4% increase in daily consultations for respiratory diseases. The study showed a delayed effect from lags 6–15, which was slower and longer than the 0–2 lags observed in our study. Our study showed that the effect of temperature among the elderly was evident on days immediately after strikes, but not on the normal strike-free days, suggesting that political strikes modified the effects of temperature on asthma diseases in this subgroup. A possible explanation would be the tendency of this subgroup to wait out the mass strike activities so they could return to the same hospital for treatment, likely because of personal preference (an existing patient at NAC, or because of NAC’s specialization in respiratory medicine), problems accessing other facilities, mobility issues, the lack of alternatives/information, or a combination of these factors. Further studies are needed to investigate issues affecting the ability of the elderly and others to seek emergency medical help during “Hartal” events. A study in Nepal reported evidence of lower maternal health care utilization in conflict-affected areas, and hence the need to decentralize services and provide community-based and mobile clinics [22].

We did not find any association between ambient temperature and emergency asthma visits among the 1067 children (≤18 years old) in this study. Previous studies of childhood asthma in Canada [14], Athens [25], and Tokyo [18] also found no association. But these are in contradiction of the findings from a Spanish study of 371 children, which reported a 7.3% increase in the monthly asthma admissions at a pediatric emergency department for each degree centigrade decrease in the monthly average temperature [16]. The association has also been reported among children in Sweden [21] and Shanghai [11]. Given the mixed results and lack of findings from warm climates, it is challenging to draw conclusions about the nature of temperature-asthma relationship in children.

A positive effect of temperature on the same day (lag 0) was observed among female adults. Further analyses should examine if this is due to the unaccounted effects of indoor dust endotoxin being ascribed to temperature, given the positive correlation of dust endotoxin and temperature [35]. Also, females who spend relatively more time indoors may be more exposed to indoor cooking pollutants which have been linked to the exacerbation of asthma symptoms [36,37]. 

Our study has a few limitations. We were unable to conduct patient interviews to obtain data for December 2013 and January 2014 because of political strikes that occurred almost every day during the two months. This means our findings should be confirmed using a longer time-series to account for cold season. Modification of temperature effect by season should also be checked. Nonetheless, our study showed that even with the warm temperatures (daily mean temperature ≥18.2 °C), a temperature decrease was associated with increased hospital asthma attendance in certain subgroups. Our work assumed a linear temperature exposure, but a flexible approach for nonlinear exposure can be useful provided a longer time series is available. Another limitation involves the omission of air pollution, which has been implicated in the exacerbation of asthma [38]. Air pollution data was not available and therefore not included in the current analysis. A separate study to determine the effect of air pollution is warranted. In the current study, we estimated the total effects of temperature without any adjustment for air pollution in view of the latter’s role as an intermediate variable on the pathway between temperature and asthma visits [13,39,40]. Lastly, like many population-based studies that rely on monitoring data, discrepancies in the true level of individual exposure may have resulted in estimation bias, which should be taken into consideration when making inferences.

## 5. Conclusions

Temperature declines in tropical Dhaka triggered asthma exacerbations in adults and the elderly. This association was not observed in children, but results should be validated using a longer study period. The frequent political strikes in the city modified this association among the elderly, who showed an increased asthma attendance associated with temperature declines on days immediately after a strike. More studies in warm climates are needed to substantiate the observed negative effects of low temperatures on asthma.

## Figures and Tables

**Table 1:** Asthma visits treated with nebulized medication at the National Asthma Centre, Mohakhali, Dhaka, Bangladesh and strike events occurring in the vicinity, February–November 2013.

Patients and Strike Events	Total n (%)	Age Group, n (%)		
		Children (≤18 Years)	Adults (19–59 Years)	Elderly (≥60 Years)
**Patients by sex**				
Male	3483 (58)	651 (19)	2127 (61)	705 (20)
Female	2506 (42)	416 (17)	1804 (72)	286 (11)
Total	5989 (100)	1067 (18)	3931 (66)	991 (16)
**Strike events**				
Total number of days	48			
Number of continuous episodes	26			
Mean number of days per episode (range)	1.8 (1–4)			
Mean number of days per month	4.8			

**Table 2:** Percentage change (95% confidence interval) in the daily number of asthma patients treated with nebulized medication in relation to a 1 °C decrease in mean temperature according to sex and age group.

Age Group	Male n = 3483			Female n = 2506			Total n = 5989		
	Change (%)	95% CI	Lag	Change (%)	95% CI	Lag	Change (%)	95% CI	Lag
Children	0.80	(−7.78, 10.19)	0–6	−2.45	(−9.77, 5.47)	0-2	−1.44	(−7.98, 5.57)	0–6
Adults	8.22	(2.81, 13.92) **	0–7	3.18	(−2.58, 9.28)	0-7	5.84	(1.87, 9.97) **	0–7
Elderly	6.38	(−0.19, 13.39) *	0–2	5.59	(−8.39, 21.71)	0-7	5.69	(−1.49, 13.39)	0–6
All ages	6.29	(2.40, 10.33) **	0–6	2.20	(−2.63, 7.28)	0-7	4.47	(1.53, 7.50) **	0–6

Note: The largest cumulative estimates (expressed as percentage changes) and the corresponding lag structures are shown. Cumulative effects were estimated using mean-constrained daily temperature and were adjusted for strikes and relative humidity. * *p* < 0.10; ** *p* < 0.05.

**Table 3:** Percentage change (95% confidence interval) in the daily number of asthma patients treated with nebulized medication in relation to a 1 °C decrease in mean temperature by the different periods affected or not affected by strikes.

Age Group	Days Not Affected by Strike * (n = 4682)			Days Immediately After a Strike (n = 633)		
	Change (%)	95% CI	Lag	Change (%)	95% CI	Lag
Children	0.69	(−6.26, 8.15)	0–6	−7.05	(−19.52, 7.35)	0–7
Adults	7.21	(3.03, 11.56) **	0–7	5.20	(−3.64, 14.84)	0–11
Elderly	5.58	(−1.75, 13.46)	0–6	15.65	(1.23, 32.12) **	0–4
All ages	5.76	(2.40, 9.22) **	0–7	3.13	(−2.16, 8.72)	0–5

The largest cumulative estimates (expressed as percentage changes) and the corresponding lag structures are shown. Cumulative effects were estimated using mean-constrained daily temperature and were adjusted for relative humidity. * Exclude days during strikes and days directly after a strike. ** *p* < 0.05.

## Data Availability

The data presented in this study are available on request from the corresponding author. The data are not publicly available due to ethical restrictions.

## Supplementary Materials

The following are available online at https://www.mdpi.com/1660-4601/18/3/890/s1, Figures S1 and S2.
