# Supplementary material for: Effect of Ambient Temperature on Daily Nebulized Asthma Hospital Visits in a Tropical City of Dhaka, Bangladesh
Source: Int J Environ Res Public Health. 2021 Jan 20;18(3):890. doi: 10.3390/ijerph18030890 (PMC7908622; doi:10.3390/ijerph18030890)
Supplement: Supplementary file 1 [file ijerph-18-00890-s001.pdf]

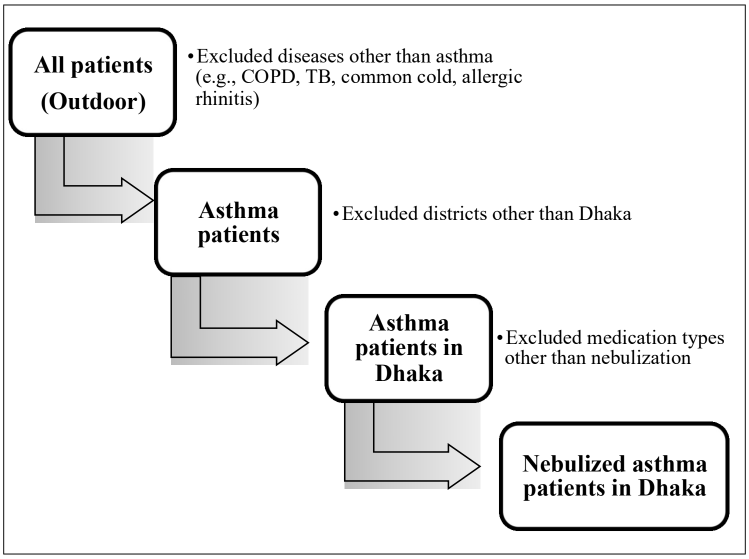

Figure S1. Flow chart for the extraction of patients' data.

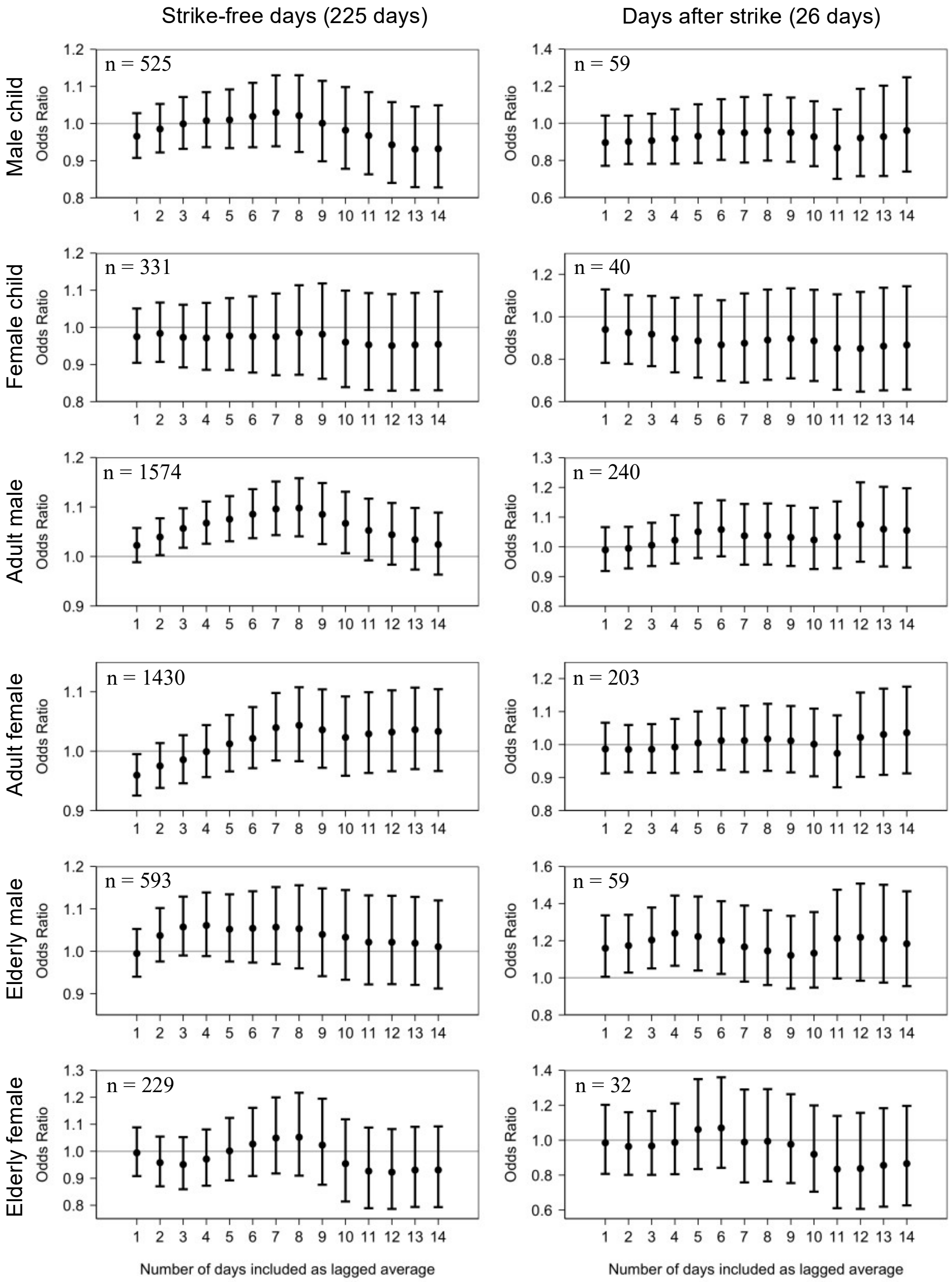

Figure S2. Adjusted odds ratios for the daily number of asthma patients treated with nebulized medication in relation to a 1°C decrease in daily mean temperature, stratified by strike event.
